# Cytological Features of a Variant NUT Midline Carcinoma of the Lung Harboring the *NSD3-NUT* Fusion Gene: A Case Report and Literature Review

**DOI:** 10.1155/2015/572951

**Published:** 2015-01-19

**Authors:** Shiho Kuroda, Shioto Suzuki, Akira Kurita, Mari Muraki, Yoichiro Aoshima, Fumihiko Tanioka, Haruhiko Sugimura

**Affiliations:** ^1^Division of Pathology, Iwata City Hospital, 512-3 Ookubo, Iwata, Shizuoka 438-8550, Japan; ^2^Division of Respiratory Medicine, Iwata City Hospital, 512-3 Ookubo, Iwata, Shizuoka 438-8550, Japan; ^3^Department of Tumor Pathology, Hamamatsu University School of Medicine, 1-20-1 Handayama, Higashi-ku, Hamamatsu, Shizuoka 431-3192, Japan

## Abstract

*Background*. Nuclear protein in testis (NUT) midline carcinoma (NMC) is a very rare and aggressive malignancy. In more than two-thirds of these NMC cases, a fusion between *NUT* and *BRD4* or *BRD3* has been documented; other variants are rare. The cytology of NMC itself has been sparsely documented and that of variant NMC has never been reported. *Case Presentation*. A 36-year-old woman was admitted because of a rapidly progressing lung tumor with metastases to the breast and bone. We recently reported this patient as the first case of a variant NMC of the lung harboring an *NSD3-NUT* fusion, based on immunohistochemical and genetic analyses. Cytological material was available for the present review. A highly cellular smear contained a predominantly noncohesive pattern of monomorphic cells with diameters 2–2.5 times greater than those of small lymphocytes, with a round-to-oval nucleus, slightly irregular nuclear contours, variably prominent nucleoli, scant cytoplasm, and identifiable mitotic figures. Foci of stratification and overt pearl formation, including a dyskeratocyte, were occasionally observed. The necrotic background contained naked nuclei, karyorrhectic debris, apoptotic cells, and macrophages phagocytizing karyorrhectic debris; nuclear crushing was noted. *Conclusion*. The cytological features of a variant NMC of the lung are described for the first time.

## 1. Introduction

Nuclear protein in testis (NUT) midline carcinoma (NMC) is a recently recognized entity that is characterized by undifferentiated morphological features and immunoreactivity to NUT [1]. This disease is a very rare [2–4] and aggressive [3, 4] malignancy that most often occurs in the midline of the body, including the head and neck and the mediastinum [2, 4]. Currently, the diagnosis of NMC depends on the identification of a rearrangement involving the* NUT* locus at 15q14 that generates a specific fusion transcript with a member of the bromodomain-containing protein (BRD) family, such as* BRD4* located on chromosome 19p13.1. In more than two-thirds of NMC cases, a gene fusion between* NUT* and* BRD4* or* BRD3* has been documented [2, 5–7]; other variant fusions are rare [6]. Recently, we described a variant NMC in which an* NSD3- (nuclear receptor binding SET domain 3-) NUT* rearrangement was identified in the primary tissue using 5′-rapid amplification of the cDNA end (RACE); the fusion was validated using fluorescence* in situ* hybridization (FISH) [8].

Little information is available on the cytological features of NMC. Three reports have described the cytological features of 4 common NMC cases [9, 10] and another NMC case in which the gene rearrangement was not analyzed [11], and no information on the cytology of variant NMC is presently available. We herein describe, for the first time, the cytological features of a variant NMC of the lung harboring an* NSD3-NUT* fusion gene.

## 2. Clinical Summary

A 36-year-old woman sought medical advice because of a cough accompanied by wheezing with a 2-month duration. An enhanced computed tomography scan performed at the time of hospitalization revealed a mass (75 × 38 × 35 mm in size) in the left lung that extended to the middle mediastinum; metastatic lesions in the liver, breast, bones, and lymph nodes were also detected [8]. A transbronchial biopsy (TBB) of the lung tumor and an endobronchial ultrasound-guided transbronchial needle aspiration (EBUS-TBNA) of a lower mediastinal lymph node with lung tumor involvement were performed.

## 3. Materials and Methods

The aspiration material obtained from the lymph node was separated into two parts; its small portion was available for cytological investigation. The specimen was air-dried and Hemacolor-stained (MERCK, Darmstadt, Germany) at bedside according to the manufacturer's instructions and a portion was fixed in 95% ethanol and stained with Papanicolaou as ordinary methods. The larger part of the aspiration material obtained from the lymph node and the biopsy material taken from the lung tumor were immediately immersed in 20% buffered neutral formalin, fixed overnight, and embedded in paraffin. These specimens were then sectioned and used for hematoxylin-eosin staining, immunohistochemistry with antibodies for EMA (DAKO, Glostrup, Denmark), p63 (Santa Cruz Biotechnology, Dallas, TX, USA), cytokeratin AE1/AE3 (DAKO), cytokeratin CAM 5.2 (Becton, Dickinson and Company, CA, USA), CD138 (DAKO), vimentin (DAKO), and others, and FISH, as reported previously [8].

### 3.1. Cytological Findings

**Figure 1:**
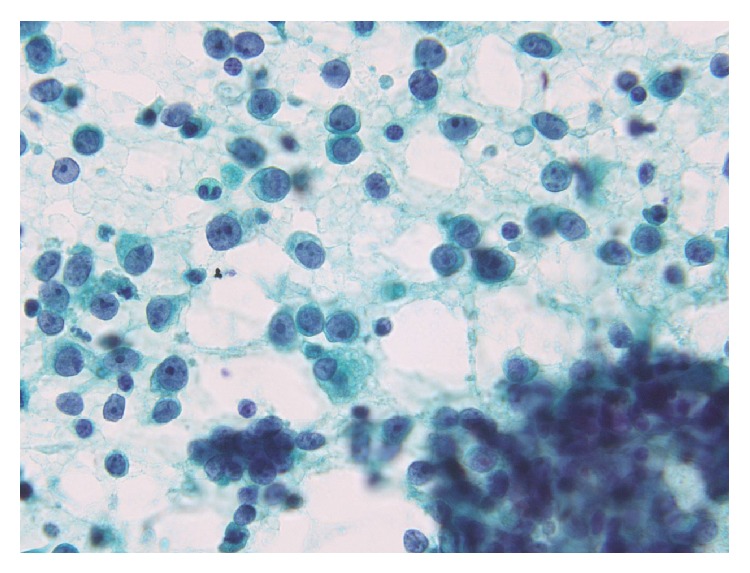
Papanicolaou-stained smear showing a high cellularity with loosely cohesive cells or isolated cells. The cells were 2–2.5 times greater in diameter than that of a small lymphocyte. The nuclei were round to oval in shape with slightly irregular contours and contained one or more prominent nucleoli. The chromatin was hyperchromatic and finely granular.

**Figure 2:**
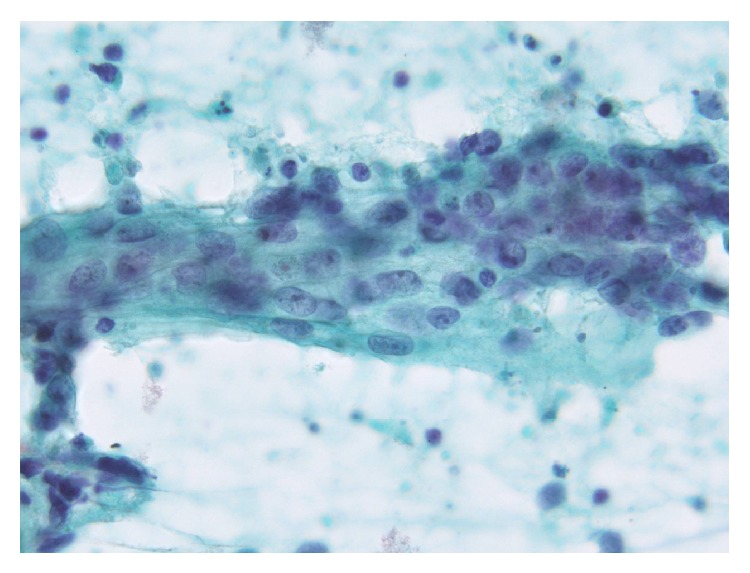
Papanicolaou-stained smear showing loosely cohesive clusters and stratification. The main cells exhibited scant cytoplasm and an indistinct cell border, forming loosely cohesive clusters (right side) that were in contact with foci of stratification (center-left side), which consisted of occasional cells with a clear cell border and moderately delicate cytoplasm.

**Figure 3:**
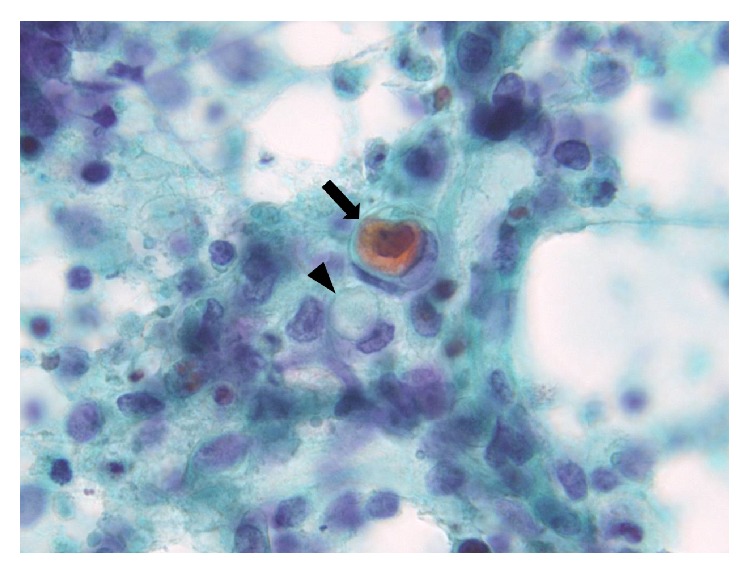
Papanicolaou-stained smear showing pearl formation. A few cells showed overt pearl formation, including a dyskeratocyte (arrow), implying keratinization. Occasional cells with moderately delicate cytoplasm and cytoplasmic coarse vacuoles (arrow head) are visible.

**Figure 4:**
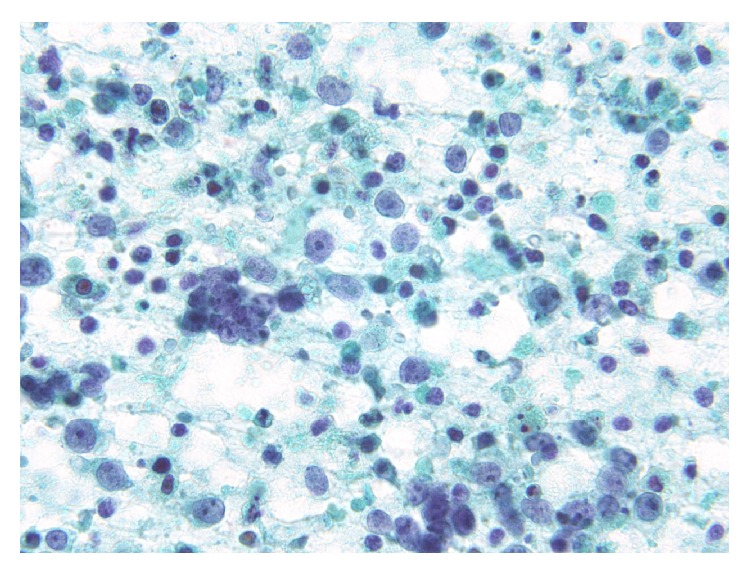
Papanicolaou-stained smear showing a necrotic background containing naked nuclei, karyorrhectic debris, apoptotic cells, and macrophages phagocytizing karyorrhectic debris.

An overview of the Papanicolaou-stained smear showed the specimen to be highly cellular with loosely cohesive cells and/or isolated cells (Figure 1). The cells were 2–2.5 times greater in diameter than that of a small lymphocyte. The nuclei were round to oval in shape and had slightly irregular contours, with one or more prominent nucleoli (Figure 1). The chromatin was hyperchromatic and finely granular in most of the cells or vesicular in occasional cells. The main cells, which had scant cytoplasm and an indistinct cell border, often formed loosely cohesive clusters (Figure 2). These clusters were in contact with foci of stratification (Figure 2), which consisted of occasional cells with a clear cell border and moderately delicate cytoplasm, often with cytoplasmic coarse vacuoles (Figure 3, arrowhead) that were negative for epithelial mucin. In addition, a few cells showed an overt pearl formation, including a dyskeratocyte (Figure 3, arrow), whereas occasional small apoptotic cells with orange G-colored cytoplasm were scattered throughout the specimen. A glandular structure was not observed. Pleomorphism of the cells was not prominent. Mitotic figures were often identified. The necrotic background contained naked nuclei, karyorrhectic debris, apoptotic cells, and macrophages phagocytizing karyorrhectic debris (Figure 4); nuclear crushing was noted.

**Figure 5:**
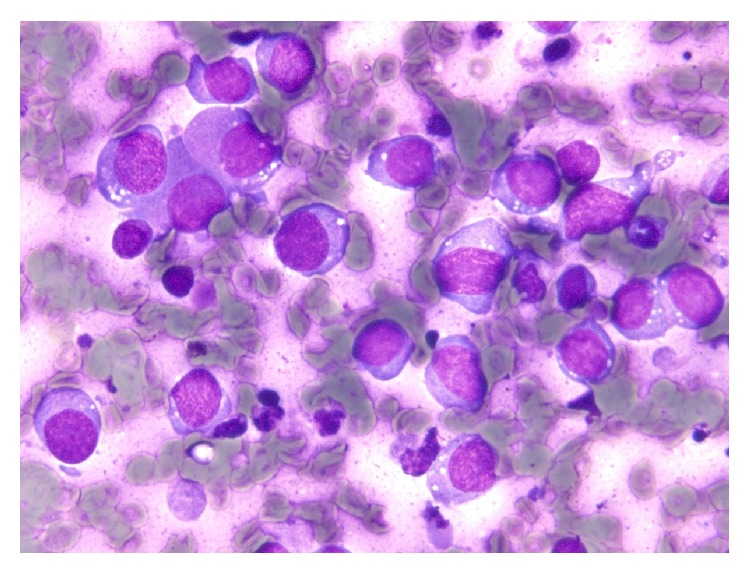
Hemacolor-stained smear showing cells bearing basophilic cytoplasm focally with several cytoplasmic fine vacuoles.

On the other hand, the Hemacolor-stained smear showed occasional cells bearing delicate basophilic cytoplasm focally with several cytoplasmic fine vacuoles (Figure 5), whereas these cytoplasmic fine vacuoles were not identified in the Papanicolaou-stained smear material.

### 3.2. Histological, Immunohistological, and FISH Findings

**Figure 6:**
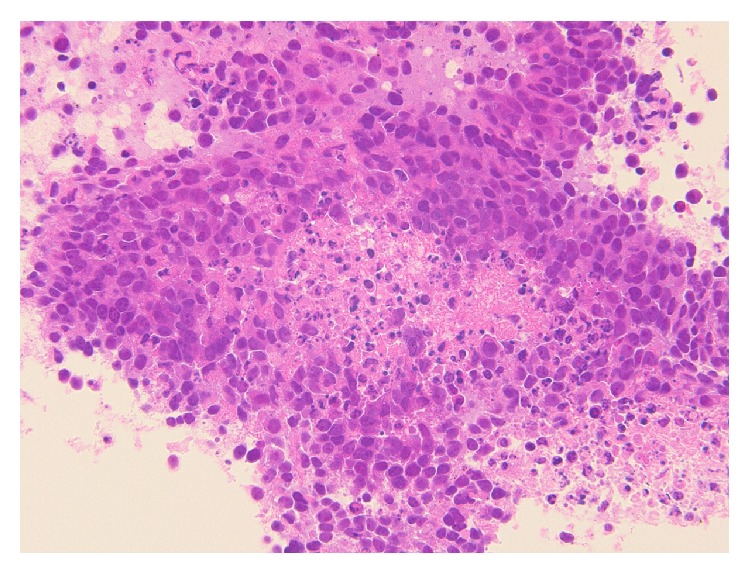
Representative histological image showing sheets of undifferentiated malignant cells with focal necrosis.

Details of the histological and molecular features of this case have been previously reported [8]. Briefly, only a small amount of biopsy was available for histological investigation, and it revealed an undifferentiated neoplasm with necrosis (Figure 6). In this material, squamous differentiation, which is a possible characteristic of variant NMC, was not apparent.

**Figure 7:**
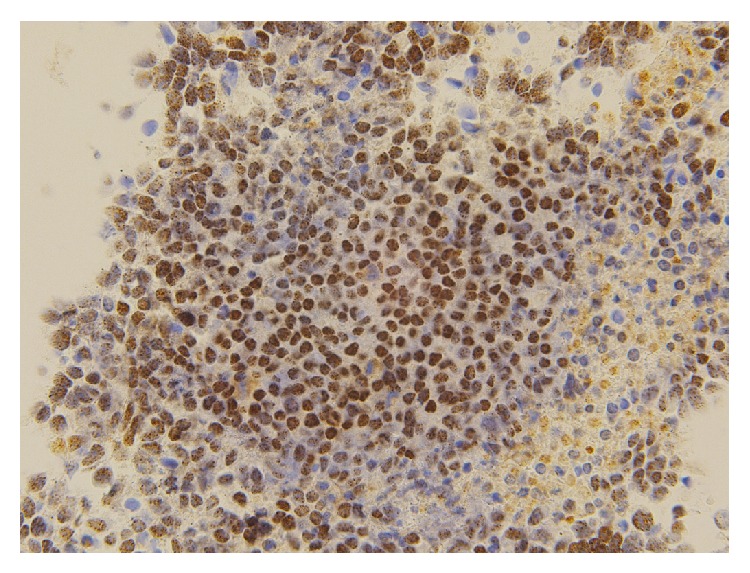
Immunohistochemistry showing a nuclear staining pattern for NUT.

**Figure 8:**
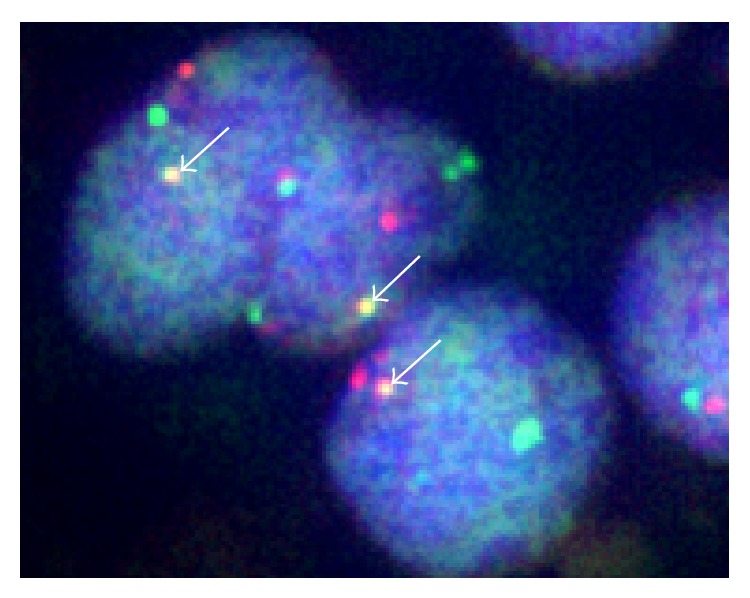
A dual-color FISH analysis showing the fusion gene as a single yellow (overlapping) signal (arrows), including a green (*NSD3*) and an orange (*NUT*) signal.

Immunohistochemical staining demonstrated focal positivity for EMA, p63, cytokeratin AE1/AE3, cytokeratin CAM 5.2, CD138, and vimentin. In addition, a nuclear staining pattern for NUT was evident (Figure 7). Furthermore, FISH analyses revealed an* NSD3-NUT *rearrangement (Figure 8), whereas* BRD3/4-NUT* fusion genes were not identified.

### 3.3. Treatment and Follow-Up

The patient received chemoradiation therapy for 10 months after her diagnosis. However, the patient died with disease progression at 10 months after the diagnosis. Autopsy was not permitted.

## 4. Discussion

The histological features of NMCs range from entirely undifferentiated carcinomas to carcinomas with prominent squamous differentiation [2, 7, 12–15]. Thus, a diagnosis of NMC based solely on morphology can be difficult. Previous studies have described the cytological features of 4 common NMCs harboring a* BRD3/4-NUT* fusion gene [9, 10] and another NMC case in which the gene rearrangement was not analyzed [11]; the cytological characteristics of these NMCs showed a highly cellular, predominantly noncohesive pattern of relatively small cells with a round nucleus, scant cytoplasm, irregular nuclear contours, variably prominent nucleoli, and identifiable mitotic figures. These findings were also observed in the present variant NMC case. Thus, the previously reported findings imply that the cytological characteristics of NMC are nonspecific and similar to those of undifferentiated carcinoma.

Keratinization has not been identified in previous studies examining the cytology of NMCs [9–11]. In theory, overt keratinization is very important and sometimes pathognomonic for a scrutinized diagnosis of NMC; that is, it is assumed to be a relatively characteristic finding of NMC harboring a* NUT* gene rearrangement involving a gene other than* BRD4* [16]. Keratinization in surgical material, however, is notorious for evading observation because its distribution is often very focal [6, 16], and sampling biases often occur. The cytology of the present NMC variant showed overt pearl formation, including a dyskeratocyte, and the stratification in contact with loosely cohesive clusters; we believe that this finding corresponds to abrupt keratinization, which has been reported as a peculiar characteristic of variant NMC [16] but was not recognized in our corresponding histology specimen [8]. Thus, the combination of cytological and histological findings may help to reveal keratinization concurrent with stratification in undifferentiated carcinoma, which is one clue for a diagnosis of NMC, especially variant NMC, rather than other tumors.

The Hemacolor-stained smear showed occasional cells possessing delicate basophilic cytoplasm with several fine vacuoles, which were not observed in our Papanicolaou-stained smear. In a previous NMC case harboring a* BRD3-NUT* fusion gene, the cells were described as possessing delicate to finely vacuolated cytoplasm, although we could not observe the cytoplasm in detail because of the low magnification of the published figures [10]. On the other hand, these cytoplasmic fine vacuoles were not observed in NMC cases harboring a* BRD4-NUT* fusion gene [9]. Further studies are needed to clarify whether these cytoplasmic fine vacuoles are specific for NMC harboring a fusion gene involving* NUT* and a gene other than* BRD4*.

In conclusion, although the distinction of NMC from other poorly differentiated carcinomas based solely on morphology is difficult, cytological investigation is helpful, especially for identifying abrupt keratinization, which histopathological investigations can miss because of sampling biases. Our experience has shown that the identification of the following clues may suggest a diagnosis of NMC; overt pearl formation including a dyskeratocyte, stratification, and cytoplasmic fine vacuoles, especially in cases where the initial suspected diagnosis was “undifferentiated or poorly differentiated carcinoma with little pleomorphism.” Furthermore, the identification of this entity is critical, and immunohistochemistry or FISH studies should be considered for the identification of* NUT* gene rearrangements.
